# Hyperammonia induces specific liver injury through an intrinsic Ca^2+^-independent apoptosis pathway

**DOI:** 10.1186/1471-230X-14-151

**Published:** 2014-08-22

**Authors:** Jingjing Li, Zujiang Yu, Qiongye Wang, Duolu Li, Bin Jia, Yubing Zhou, Yanwei Ye, Shen Shen, Yanfang Wang, Shasha Li, Lu Bai, Quancheng Kan

**Affiliations:** 1 Department of Gastroenterology, The First Affiliated Hospital of Zhengzhou University, Zhengzhou, China; 2 Department of Infectious Diseases, The First Affiliated Hospital of Zhengzhou University, Zhengzhou, China; 3 Department of Pharmacy, The First Affiliated Hospital of Zhengzhou University, Zhengzhou, China; 4 Department of Gastrointestinal Surgery, The First Affiliated Hospital of Zhengzhou University, Zhengzhou, China

**Keywords:** Liver injure, Hyperammonia, Calcium overload, Mitochondrial damage

## Abstract

**Background:**

Numerous pathological processes that affect liver function in patients with liver failure have been identified. Among them, hyperammonia is one of the most common phenomena.The purpose of this study was to determine whether hyperammonia could induced specific liver injury.

**Methods:**

Hyperammonemic cells were established using NH_4_Cl. The cells were assessed by MTT, ELISA, and flow cytometric analyses. The expression levels of selected genes and proteins were confirmed by quantitative RT-PCR and western blot analyses.

**Results:**

The effects of 20 mM NH_4_Cl pretreatment on the cell proliferation and apoptosis of primary hepatocytes and other cells were performed by MTT assays and flow cytometric analyses. Significant increasing in cytotoxicity and apoptosis were only observed in hepatocytes. The cell damage was reduced after adding BAPTA-AM but unchanged after adding EGTA. The expression levels of caspase-3, cytochrome C, calmodulin, and inducible nitric oxide synthase were increased and that of bcl-2 was reduced. The Na^+^-K^+^-ATPase activities in hyperammonia liver cells was no signiaficant difference compaired with the control group, but was decreased in astrocytes. NH_4_Cl pretreatment of primary hepatocytes promoted the activation of mitochondrial permeability transition pores and the mitochondria swelled irregularly.

**Conclusions:**

Hyperammonia induces specific liver injury through an intrinsic Ca^2+^-independent apoptosis pathway.

## Background

Acute liver failure is a multisystem disorder associated with acute renal failure, hypotension, sepsis, coagulopathy, encephalopathy, and cerebral edema [1]. Some researchers have considered that a few hepatocytes are required to restore the liver mass after profound liver injury, while other liver-repopulation and transplantation studies have indicated that bone-marrow stem cells might have the capacity to differentiate into hepatocytes [2-5]. However, the regenerative capacity is insufficient after chronic liver injury [6]. Currently, most researchers consider that the activation, proliferation, migration, differentiation, and survival of cells in the regenerating liver are controlled by a large number of growth factors and cytokines, which expressed at the sites of injury or reach the liver via the circulatory system [7]. Furthermore, a recent study found that severe lactic acidosis was harmful for cirrhotic patients [8]. Our preclinical studies showed that the clinical symptoms of some patients with liver failure, who did not present with symptoms of hepatic encephalopathy (HE) and were treated with blood ammonia-lowering drugs, were greatly relieved. The primary disease process in the liver is complicated because of numerous metabolic disturbances throughout the body [9]. Therefore, we inferred that a reduction in the blood ammonia level could promote the functions of synthesis, secretion, and transformation in liver cells and simultaneously relieve the damage to liver cells. Recent studies showed that increasing ammonia concentrations had deleterious effects on the functions of the central nervous system and the elevation of arterial ammonia was associated with high mortality in patients with acute liver failure [10,11]. Ammonia is a neurotoxin involved in the pathogenesis of neurological disease associated with hyperammonia [12]. Hyperammonia following acute and chronic liver diseases may lead to HE, which is accompanied by the failure of energy metabolism [13], disturbances of neurotransmission in the brain, and changes in Na^+^-K^+^-ATPase [14,15]. Moreover, our study showed that there was no change in Na^+^-K^+^-ATPase using gene chip assays, but arginine disappeared. Although the liver can convert ammonia to nontoxic urea through the urea cycle [16], the urea synthesis capacity is reduced in patients with liver disease, leading to a reduced capacity to detoxify ammonia in the liver. Besides, hyperammonia is also produced in urea cycle disorders and other conditions leading to either defective ammonium removal or overproduction of ammonium beyond the capacity of liver clearance [17]. Therefore, we thought that ammonia might induce liver injury through another mechanism. However, there are few reports about whether increasing blood ammonia can lead to the damage of hepatocyte function observed in the present study. We also found that NH_4_Cl induced specific liver injury compared with other cell types and apoptosis of primary hepatocytes was significantly increased compared with control cells. In this study, we hypothesized that hyperammonia might directly induce a series of changes leading to liver injury. To verify this assumption, a hyperammonia cell model was established to investigate the effects of NH_4_Cl on liver damage and further examine the effects of NH_4_Cl on hepatocyte apoptosis.

## Methods

### Cell lines, cell culture, and NH_4_Cl treatment

Primary hepatocytes were purchased from the Type Culture Collection of the Chinese Academy of Sciences (Shanghai, China). A-549 lung cancer cells, MCF-7 breast cells, BGC-823 gastric cancer cells, SKOV3 ovarian cancer cells, C6 glioma cells, and HepG2 HepG2.2.15 hepatocarcinoma cells were all preserved in our laboratory. All cells were maintained in RMPI1640 medium supplemented with 10% fetal bovine serum, 100 U/ml penicillin, and 100 μg/ml streptomycin at 37°C under 5% CO_2_. The cells were treated with NH_4_Cl at 0.5, 5, 10, and 20 mmol/L.

### Enzyme levels in media

The supernatant was extracted after exposed to 0 and 20 mM NH_4_Cl for 12, 24 and 48 h respectively. The detection of each indicator was carried out in accordance with the requirements of the ELISA kit. The OD values were detected at 450 nm with a Microplate reader (MultiskanMK3).

### Viability measurement

Viability was tested by using MTT (3-[4, 5-dimethyldiazol-2-yl]-2, 5–diphenyl -tetrazolium bromide) [18]. After different concentrations of NH_4_CL exposure, MTT was added to a final concentration of 0.5 mg/ml. After 4 h MTT-incubation, supernatants were removed and 150 μL DMSO was added in each well, darkly shaken for 15 min. The absorption was measured by a microplate reader at a wavelength of 490 nm.

### Apoptosis of different cell lines evaluated by flow cytometry

Cells were treated with NH_4_Cl, harvested after 24 hours and suspended in annexin V-binding buffer. Thereafter, the cells were incubated by FITC for 15 min in the dark, and propidium iodide was added. Thereafter, all samples were analyzed by a FACSCalibur flow cytometer with CellQuest software.

### Assay of Na-K ATPase specific activity

Total membrane fractions were prepared from control and NH_4_Cl-treated hepatocytes and C6 cells. Reaction mixtures for Na-K-ATPase activity assay was as the manufacturer’s instructions (Jianchen Biology Engineering Institute, China). Enzymatic activity was measured as a function of liberated inorganic phosphate (P i) by the colorimetric reaction. The color developed after 10 min at 37°C and was read at a wavelength of 850 nm with a spectrophotometer (SpectraMax 190, Molecular Devices, Sunnyvale, CA).

### Arginine levels after primary hepatocyte injury induced by NH_4_Cl

Primary hepatocytes were treated with 10 mmol/L NH_4_Cl for 24 h. We added 100ul sample cell fluid 350 μl extraction liquid (V_methanol_: V_chloroform_ = 3:1) and 50 μl L-2-Chlorophenylalanine as an internal standard and vortexed for 10 s. The samples were centrifuged for 10 min at 12,000 rpm and 4°C. Then we transferred 0.35 mL from the supernatant into a fresh 2 mL GC/MS glass vial. Next 80 mL methoxyamination reagent was added and shaken for 2 h at 37°C, followed by addition of 0.1 mL BSTFA reagent and shook for 1 h at 70°C. GC-MS analyses were performed when the temperatures cooled to room temperature.

### Effects of BAPTA-AM and EGTA on primary hepatocyte injury induced by NH_4_Cl

Primary hepatocytes seeded in 96-well plates were treated with media containing various concentrations of NH_4_Cl for 1 h, followed by addition BAPTA-AM (1 × 10^-5^ mol/L). Next, NH_4_Cl and EGTA were added simultaneously. The control group was treated with solvent alone. MTT solution was added after BAPTA-AM for 6 h. Absorbance was measured at 450 nm using a microplate reader.

### Detection of mitochondrial permeability transition pore (mPTP) opening

Primary hepatocytes were seeded in 48-well plates. After treatment with 20 mmol/L NH_4_Cl for 24 h, 500 μl GENMED reagent A preheated in 37°C was added to each well and then removed reagent A carefully. Then add 200 μl dyeing working fluid (5 μl reagent B plus 500 μl reagent C) was added to each well incubating at 37°C in cell culture box for 20 min darkly. Next we wash the cells with liquid A twice. Then images were collected as described. The cells were observed by fluorescence microscopy using an excitation wavelength of 488 nm and an emission wavelength of 505 nm. The peak shifting to the left indicated the increased activity of MPTP pore.

### Analysis of mitochondrial morphology by transmission electron microscopy

Primary hepatocytes were treated with 0, 10, and 20 mM NH_4_Cl for 48 h. Then the cells were harvested, fixed in 2.5% glutaraldehyde, and embedded in propylene oxide and epoxy resin overnight at 37°C. The embedded cells were cut into ultrathin sections, double-stained with uranyl acetate and lead citrate, and observed by transmission electron microscopy.

### RNA extraction and quantitative real-time PCR

Total RNA was extracted from primary hepatocytes after 12, 24, and 48 h of NH_4_Cl treatment using the TRIzol reagent (Takara, Japan). First-strand cDNA was synthesized from 1 μg total RNA using a PrimeScript RT Reagent Kit With gDNA Eraser. The cDNA was used to detect the expression levels of caspase-3, cytochrome C (Cyt C), calmodulin, and inducible nitric oxide synthase (iNOS). Quantitative real-time PCR was performed using SYBR Premix Ex Taq II in a Step One Plus system.

### Western blot analysis

To extract the total proteins, livers or cells which were treated with NH_4_Cl were lysed on ice for 30 min in lyses buffer [19], and centrifuged at 12000 g for 10 min, the supernatant were recovered. After denaturation, 50 μg proteins were separated on 10% SDS/PAGE gels and then transferred to nitrocellulose membranes by using a transfer cell system (Bio-Rad, California, USA). Membranes were blocked for 1 h at room temperature with 5% nonfat dried milk powder/Tris-buffered saline Tween-20 (TBST) and then with primary antibodies incubated overnight at 4°C. Immunoblots were washed 3 times with TBST and were incubated with secondary antibodies conjugated with horseradish peroxidase against mouse IgG or rabbit IgG for 1 h at room temperature. Immunoreactive proteins were visualized using the infrared laser scanning imaging system (CDYSSEY CLx; General Electric Company).

### Statistical analysis

Statistical analysis was performed with SPSS 13.0 software (SPSS Inc., Chicago, IL, USA). Data are expressed as means ± SD, and three individual experiments were carried out in triplicate. The Student’s t-test was used to compare data between two groups. One-way ANOVA and Dunnett’s test were used to compare data between three or more groups. P < 0.05 was considered to indicate a statistically significant result.

## Results

### Hyperammonia induces specific inhibition of liver regeneration

#### Liver damage caused by ALT and AST in vitro

**Figure 1:**
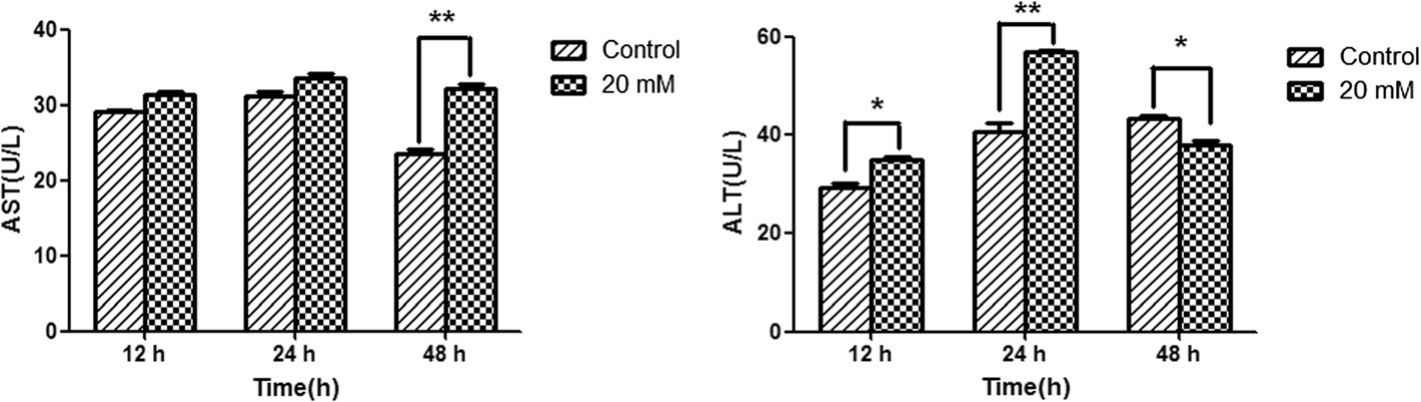
**Liver damage caused by ALT and AST in vitro.** Values are expressed Mean ± SD, *P < 0.05 versus control group, **P < 0.001 versus control group. ALT: glutamate pyruvate transaminase; AST: Aspartate aminotransferase.

The expression of ALT, markers of liver injury, (Control group: 43.44 ± 0.70, 20 mM ammonia group: 37.87 ± 0.91) was in a relatively decreased within 48 h (Figure 1A). Significantly increased expression AST (Control group: 23.59 ± 0.51, 20 mM ammonia group: 32.04 ± 0.66) was detected only at the time of 48 h (Figure 1B).

#### Effects of NH_4_Cl on cell growth in cell lines

**Figure 2:**
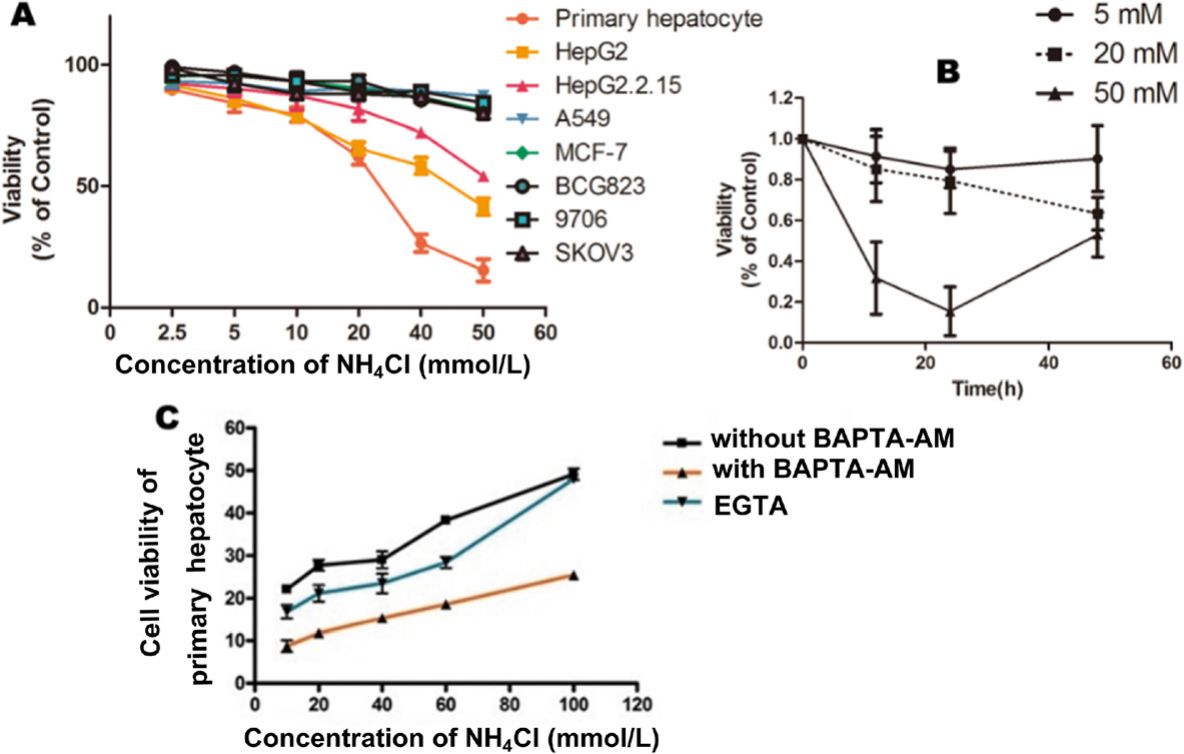
**Effects of NH**_**4**_**Cl on the cytotoxicity of primary hepatocytes and different cell lines.** Cytotoxicity was measured by MTT assays. **(A)** The levels of MTT reduction were quantified and compared with the control at each concentration. The results showed that NH_4_Cl induced specific cytotoxicity toward regenerating hepatocytes. **(B)** The viability of primary hepatocytes after exposured to 5, 20 and 50 mM ammonia for 48 h. A dramatic time-dependent loss was found in cell viability when exposure to 20 mM and 50 mM ammonia, however the viability of cells recovered when exposed to 5 mM ammonia for 48 h. **(C)** Effects of BAPTA-AM and EGTA on primary hepatocyte injury induced by hyperammonia. The cell viability of the hyperammonia group without BAPTA-AM or EGTA was clearly lower than those in the groups with BAPTA-AM or EGTA addition. Each value indicates the mean ± SEM normalized by the control for each concentration. Data were obtained from three independent experiments.

The MTT assay showed that the liver cells was inhibited by NH_4_Cl (Figure 2A), but had no effect on A549, MCF-7, BCG823, SKOV3. At 24 h, we determined that primary hepatocytes (IC_50_: 27.26 mmol/L) were significantly more sensitive to NH_4_Cl than HepG2 cells (IC_50_: 43.00 mmol/L) or HepG2.2.15 cells (IC_50_: 62.78 mmol/L). A dramatic time-dependent loss was found in cell viability when exposure to 20 mM and 50 mM ammonia, however the viability of cells recovered when exposed to 5 mM ammonia for 48 h (Figure 2B). Considering these results, we decided to use the 20 mM ammonia for the following experiments.

#### NH_4_Cl-induced apoptosis in primary hepatocytes

**Figure 3:**
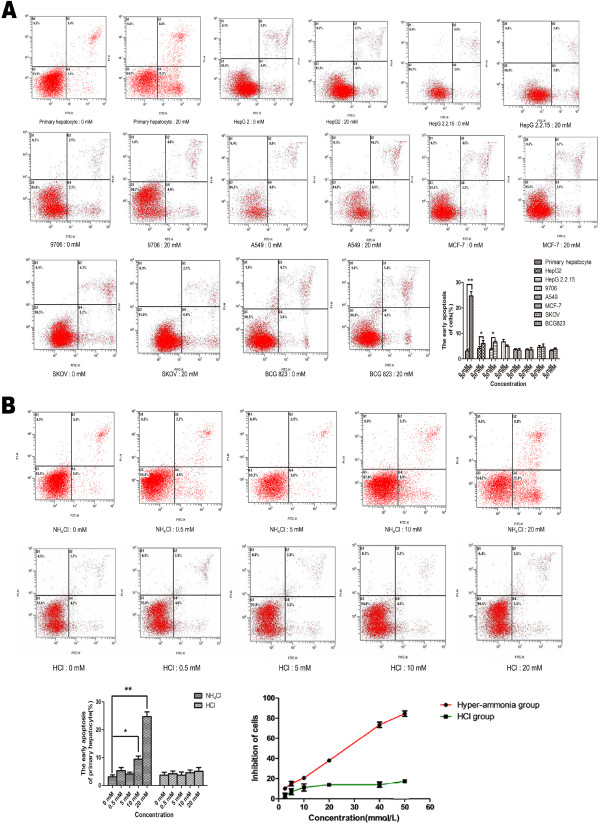
**Flow cytometry analysis of apoptosis in different cell lines.** Flow cytometry was used to assess changes in the rate of apoptosis in the primary hepatocytes and other different cell lines with NH_4_Cl treatment. **(A)** The results showed that the apoptosis rates in the hyperammonia group of primary hepatocytes (24.7 ± 1.71) were significantly greater than those in the control group (3.1 ± 0.71). while The hyperammonia groups of HepG2.2.15 (0 mM 3.73 ± 0.47; 20 mM: 6.60 ± 0.36) and HepG2 cells (0 Mm: 4.07 ± 0.76; 20 mM: 6.03 ± 0.76) showed increased rates, but they were less than those in the primary hepatocytes. The apoptosis in the other cell lines with hyperammonia (9706: 0 mM 6.7 ± 0.91; 20 mM: 4.9 ± 0.51; A549: 0 mM 3.63 ± 0.40; 20 mM: 3.40 ± 0.70; MCF-7: 0 mM 3.57 ± 0.35; 20 mM: 3.53 ± 0.86; SKOV3: 0 mM 4.47 ± 0.75; 20 mM: 4.60 ± 1.23; BCG-823: 0 mM: 3.2 ± 0.36; 20 mM: 3.83 ± 0.45) showed no difference. **(B)** Flow cytometry revealed that the apoptotic hepatocytes in the hyperammonia group were increased compared with the control group, especially when the concentrations of NH_4_Cl were 0.5 and 20 mmol/L. In addition, there were no significant differences among the HCl groups. For all experiments, each value indicates the mean ± SEM normalized by the control. *p < 0.05; **p < 0.01, one way ANOVA.

As shown in Figure 3A, the apoptosis assays evaluated by flow cytometry demonstrated that the apoptosis rate was increased to 25.97 ± 1.46% in primary hepatocytes after treating with 20 mmol/L NH_4_Cl for 24 h compared with that in the control group, which exhibited approximately 3.30 ± 0.44% spontaneous apoptosis.

#### Roles of caspase-3, Cyt C, and bcl-2 in liver regeneration

**Figure 4:**
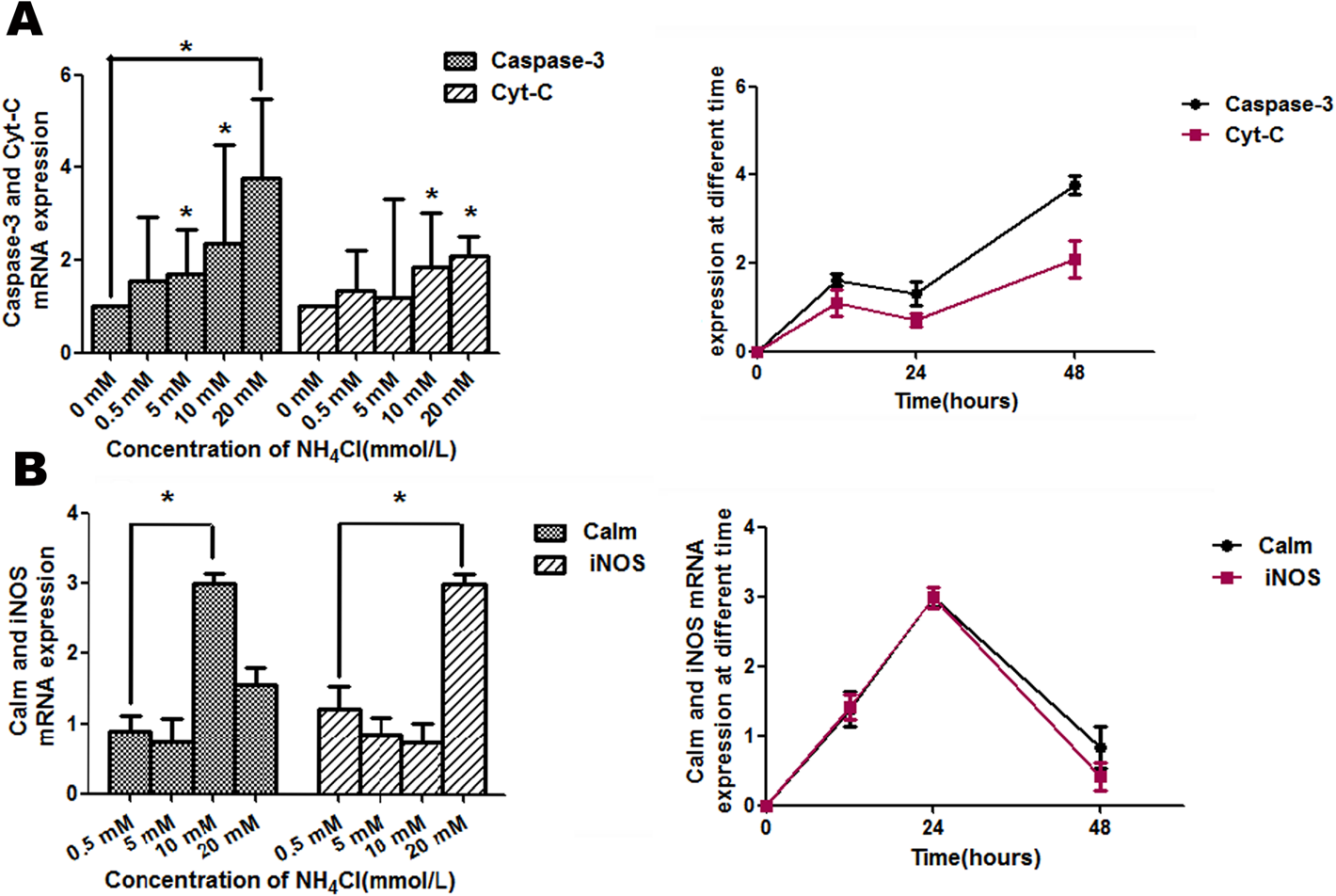
**Analysis of mRNA levels for caspase-3, Cyt C, calmodulin and iNOS, in primary hepatocytes.** Real-time PCR analyses were performed to detect the mRNA expression levels of caspase-3 and Cyt C **(A)**, calmodulin, iNOS **(B)** in liver cells following treatment with NH_4_Cl for 12, 24 and 48 h. The data were obtained from three independent experiments. For all experiments, each value indicates the mean ± SEM normalized by the control for each time point and concentration. *p < 0.05; **p < 0.01.

**Figure 5:**
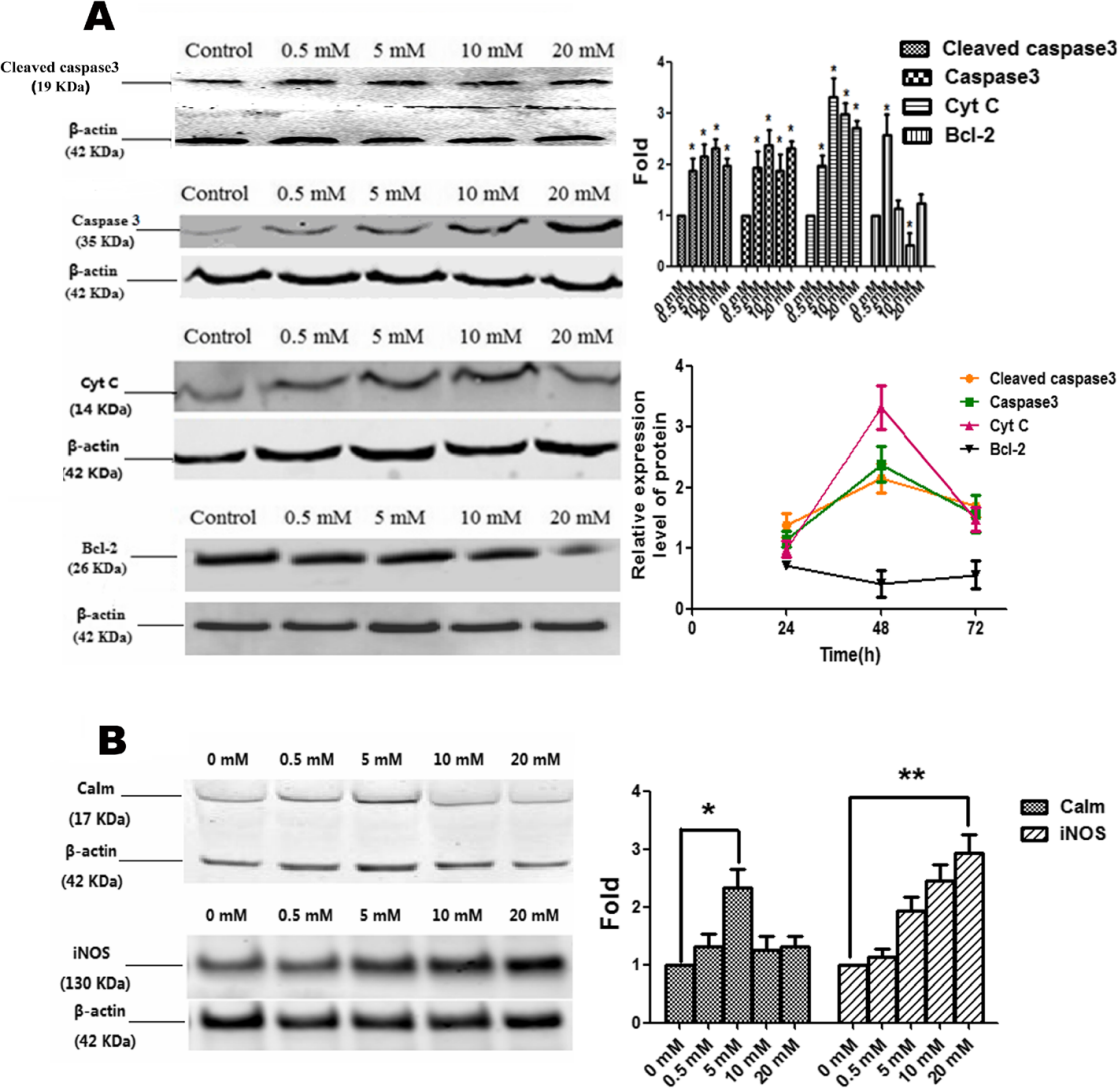
**Effect of NH**_**4**_**Cl on the expressions of cleaved caspase-3, caspase-3, Bcl-2, Cyt C, calmodulin, and iNOS.** Ammonia-induced apoptosis of liver cells was accompanied by increased levels of cleaved caspase-3, caspase-3 and Cyt C and a decreased level of bcl-2 **(A)**, and these phenomena were clearly observed after treating with NH_4_Cl for 48 h. To clarify the mechanism of the apoptosis, the protein levels of calmodulin and iNOS **(B)** were detected by western blotting analyses and all of these levels were increased. For all experiments, each value indicates the mean ± SEM normalized by the control for each time point and concentration. *p < 0.05; **p < 0.01.

**Figure 6:**
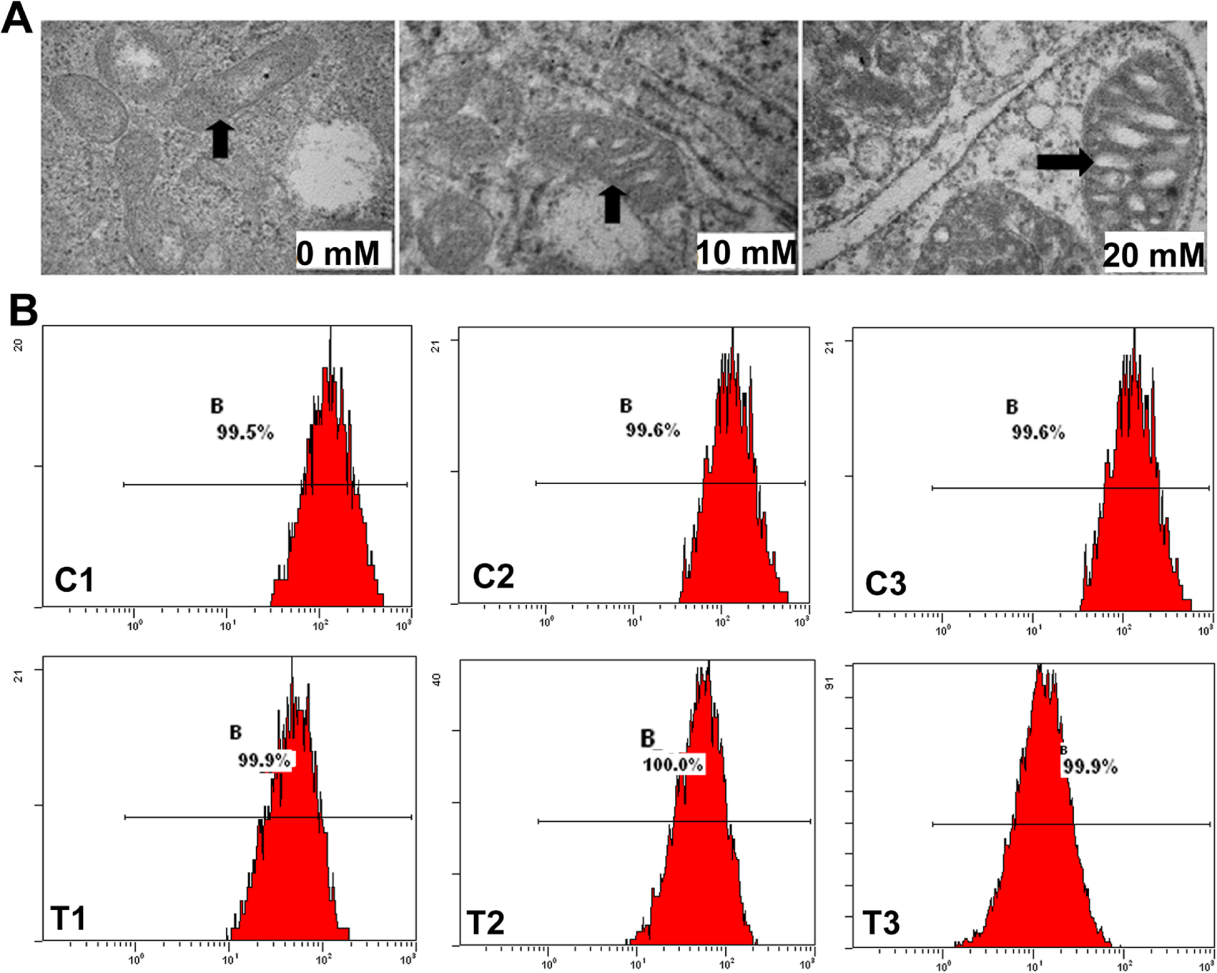
**Analysis of mitochondrion in primary hepatocytes. (A)** The morphologic features of the mitochondrion were observed by transmission electron microscopy. The changes in mitochondria were dependent on the concentration of ammonia exposure. We observed that the mitochondria showed irregular swelling, with fractured cristae and vacuolar degeneration. **(B)** C1: The position of peak shift in the control group at 0 min. C2: The position of peak shift in the control group at 15 min. C3: The position of peak shift in the control group at 30 min. T1: The peak shift position of the primary hepatocyte group with 20 mmol/L NH_4_Cl at 0 min. T2: The peak shift position of the primary hepatocyte group with 20 mmol/L NH_4_Cl at 15 min. T3: The peak shift position of the primary hepatocyte group with 20 mmol/L NH_4_Cl at 30 min. The position of peak shift in the same group gradually moved to left with the passage of time, while at the same period of time, the position of peak shift in the hyperammonia group moved to left more significantly than that in the control group.

To examine whether hyperammonia induces liver injury through an apoptosis pathway, we examined the effects of NH_4_Cl on apoptosis by measuring the levels of caspase-3, Cyt C, and bcl-2. The results of western blot are consistent with QT-PCR. The energy barrier caused by ammonia increased the expression of caspase-3, Cyt C, and accompanying reduced bcl-2 expression (Figures 4 and 5A). As depicted in Figures 5 and 6A, the mRNA expression of caspase-3 and Cyt C were significantly increased after NH_4_Cl treatment, which exhibited maximal extent when treated with 20 mmol/L NH_4_Cl at 48 h. The protein expression were significantly changed after treating with NH_4_Cl for 48 h and then returned to the control levels at 72 h. The results showed that the expression of caspase-3 (2.18 ± 0.31) and Cyt C (0.55 ± 0.14) were most increased when the concentration of NH_4_Cl was 10 mmol/L, while the expression of bcl-2 (0.55 ± 0.14) was most significantly decreased at 48 h.

### Differences in liver damage induced by hyperammonia between liver cells and other cells

#### Activity of Na-K ATPase

To elucidate the differences in Na-K-ATPase activity between liver cells and astrocytes, we measured the Na-K-ATPase activity in membrane fractions of liver cells and astrocytes treated with 20 mM NH_4_Cl. In the liver cells, the Na-K-ATPase activity was no significant difference (control group: 11.4 ± 4.5 nmol Pi/μg protein/min NH_4_Cl-treated group 12.8 ± 2.9 nmol Pi/μg protein/min). However, in the astrocytes, the Na-K-ATPase activity was significantly increased in NH_4_Cl-treated group (control group: 13.9 ± 3.6 nmol Pi/μg protein/min NH_4_Cl-treated group: 47.6 ± 2.9 nmol Pi/μg protein/min).

#### Arginine level after primary hepatocyte injury induced by NH_4_Cl

Compared with the control group, the arginine level was decreased in the NH_4_Cl-treated group (0.0003023 ± 0.3271), representing 69.16 ± 0.4179% of the arginine level in the control group (0.0004372 ± 0.2146).

### Hyperammonia induces mitochondrial damage

#### Hyperammonia induces the change of morphologic features of mitochondria

To investigate the mechanism of the liver damage induced by hyperammonia, we examined the morphological features of mitochondria by transmission electron microscopy. We observed the changes in the mitochondria depending on the ammonia exposure with 10 and 20 mmol/L. The mitochondria showed irregular swelling, fractured cristae, and vacuolar degeneration (Figure 6A).

#### Effects of BAPTA-AM and EGTA on primary hepatocyte injury induced by hyperammonia

We observed that the cell viability was increasd (51.83 ± 2.30%) after BAPTA-AM treatment (Figure 2C) compaired with the control group (76.51 ± 1.96%) which was only treated with 100 mmol/L NH_4_Cl. Each value is shown as the mean ± SEM, after normalization by the control for each concentration. In contrast, the cell viability of primary hepatocytes was unchanged after EGTA addition.

#### Hyperammonia triggers mPTP opening

Treatment of hepatocytes with 20 mM ammonia (48 h) caused the position of peak shift move to left, but the position of peak shift has little change in control group(as shown in Figure 4B: C1 and T1). Comparison of C1 and C2, the position of peak shift move to left enhanced membrane channel activity (Figure 6B: T1, T2 and T3).

### Hyperammonia induces apoptosis through an intrinsic Ca^2+^-independent apoptosis pathway

We examined the effects of NH_4_Cl on apoptosis through an intrinsic Ca^2+^-independent apoptosis pathway by measuring levels of calmodulin and iNOS. The results of WB are consistent with QT-PCR. The energy barrier caused by ammonia increased the expression of calmodulin and iNOS (Figure 5B). As depicted in Figures 4 and 5B, the mRNA expression of the molecules did not change after treatment with NH_4_Cl for 12 h (p > 0.05; data obtained from three independent experiments) and were significantly increased after treating with 20 mmol/L NH_4_Cl for 24 h. The protein expressions were significantly changed after NH_4_Cl treatment for 48 h and then returned to the control levels at 72 h. The results showed that the most significantly increased expressions of calmodulin (2.34 ± 0.31) and iNOS (2.46 ± 0.14) were observed after treatment with NH_4_Cl at 10 mmol/L for 48 h.

## Discussion

Numerous pathological processes which affect liver function in patients with liver failure have been identified. Among them, hyperammonia is one of the most common phenomena. It is well known that many growth factors and cytokines have explicit effects on regulating liver regeneration [7]. However, it remains unclear whether hyperammonia has an effect on liver injury. Currently, most researchers believe that ammonia is a kind of toxicant which can inhibit many cell functions, such as cell regeneration. In our studies, we showed that hyperammonia could induce specific liver injury, while liver damage could elevate the concentration of blood ammonia.

In agreement with previous work [20,21], our study demonstrated that NH_4_Cl induced liver damage, resulting in considerable cytotoxicity, and showed that the ALT and AST levels were increased because of the cytotoxicity against regenerating hepatocytes, which are biomarkers of pathological changes in the liver [22]. We also investigated the toxicity of ammonia to hepatocytes. With the increased concentration and time of ammonia exposed to hepatocytes, significant differences were found in the cell viability, indicating hepatocytes were sensitive to ammonia toxicity (Figure 2A and B). Many studies have focused on the neurotoxicity of ammonia, however, ammonia is not toxic to all cells. Hassel T et al. [23] found different cells show different growth inhibition when exposed to the same concentrations of ammonia. It suggested that the effect of ammonia on cells has significant cell specificity. Our study demonstrated only hepatocytes were inhibited, as evaluated by exposing various cell lines to NH_4_Cl. Furthermore, the apoptosis rates in the hyperammonia group of primary hepatocytes were significantly higher than those in the control group, especially when the concentrations of NH_4_Cl were 20 mmol/L. However, the apoptosis rates for hyperammonia of other cell lines showed no differences. To eliminate the influence of the pH value in our study, we adjusted the different NH_4_Cl concentrations with dilute hydrochloric acid solution to the same pH values.We found that the pH value had no effects on the cytotoxicity and apoptosis (Figure 3B).

Investigators now believe that hyperammonia is a major factor in HE, which is associated with liver disease [24]. HE represents a broad continuum of neuropsychological dysfunctions in patients with acute or chronic liver disease. The pathophysiology of the disease is complex, which involves overproduction and reduced metabolism of various neurotoxins, particularly ammonia [25]. Currently, there are different theories about HE [26,27], and the majority of studies have focused on ammonia poisoning. In the present study, we found that hyperammonia could lead to different expression of some important genes, such as glucose transporter (GLUT-1), glycine transporter (GLYT-1), Na^+^-K^+^-ATPase [28,29]. However, in our previous study, we found that Na^+^-K^+^-ATPase showed no difference under hyperammonia conditions in gene chip assays, while arginine was decreased [30]. Furthermore, we found that the activity of Na^+^-K^+^-ATPase in hepatocytes under hyperammonia did not significantly differ from the control level, but it was decreased in astrocytes. Therefore, we consider that the mechanism of the liver damage induced by hyperammonia differs from that in HE.

To investigate the mechanism of the liver damage induced by hyperammonia, we measured the mitochondrial in hepatocytes. With increasing concentration of ammonia, the morphologic of mitochondrion began to be abnormalities (swelling and turn round, Crista disorder, vacuolar degeneration) (Figure 6A). To further examine the function of mitochondrion, we measured the mPTP by calcein fluorescence, we found that ammonia induced opening of mPTP after exposed to ammonia (Figure 6B). The mPTP, which represents the increaseing in the permeability of mitochondrion, is a protein pore which is formed in the inner membrane of the mitochondria under certain pathological conditions [31,32].

**Figure 7:**
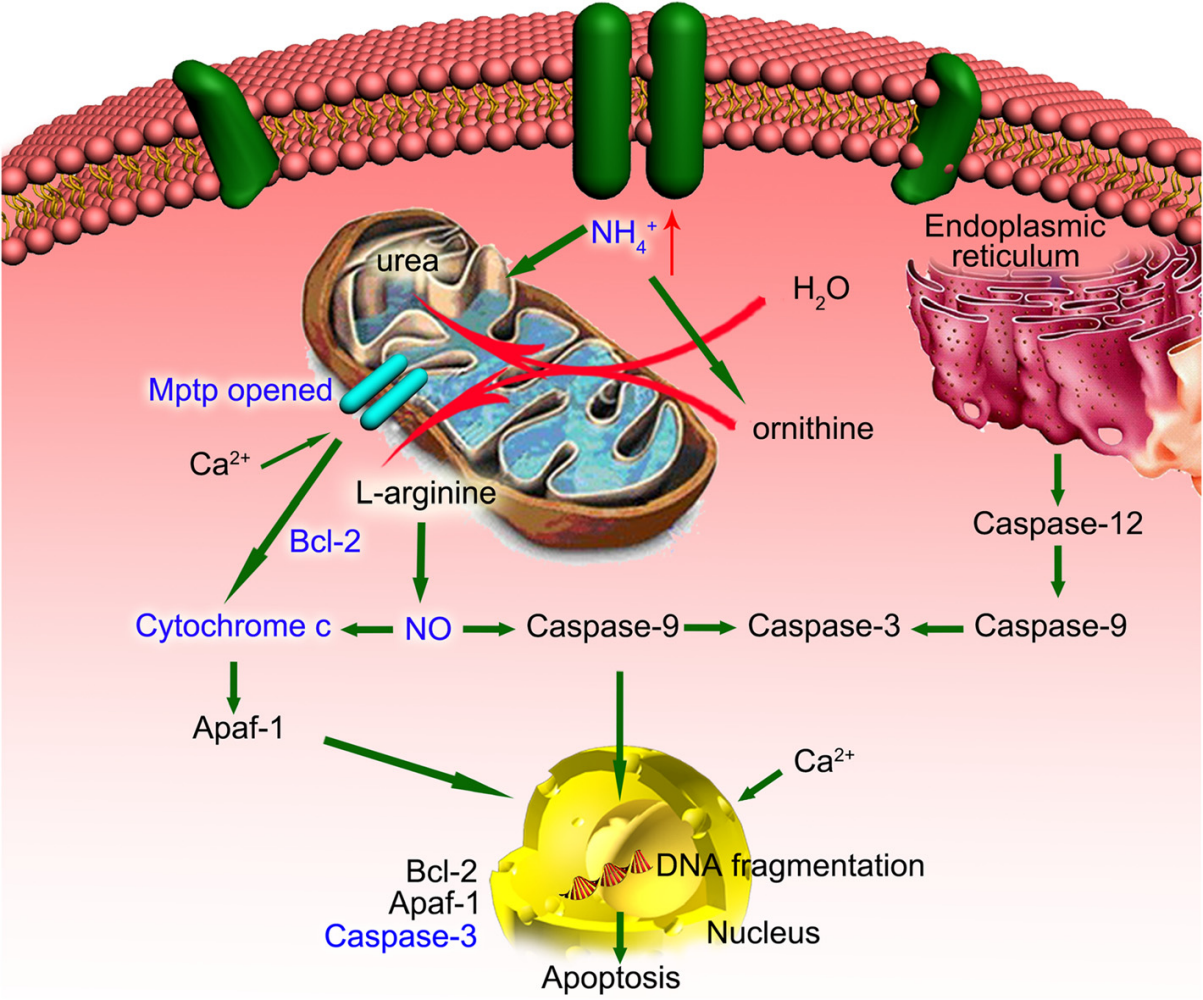
Apoptotic pathway caused by the cytochrome.

MPTP was opened when Ca^2+^ was transferred into the mitochondria and Cyt C was released [33-35]. To further confirm the hyperammonia affecting the dysfunction of mitochondrion in hepatocytes, we found that the cytotoxicity of NH_4_Cl toward hepatocytes was significantly reduced when adding BAPTA/AM, which is an effective intracellular Ca^2+^ chelator [36]. However, the cytotoxicity was unchanged by adding EGTA, which can only bind to extracellular calcium. To confirm the results, we detected the expression levels of calmodulin and iNOS by real-time PCR and western blotting analyses. We found that both of these molecules were increased, especially for treatment with 10 mmol/L NH_4_Cl. Moreover, we found arginine was decreased. It is well known that arginine is the direct precursor of nitric oxide (NO) and urea [37]. NO may be important as a signaling factor in many cells [38], and plays a role in apoptosis. A high concentration of NO, which is produced from iNOS (Figure 7), has been shown to suppress cell proliferation and induce cell apoptosis through Ca^2+^/calmodulin-dependent pathway [39]. To validate this view, we detected the expression levels of calmodulin and iNOS by real-time PCR and western blotting analyses in hepatocytes under hyperammonia, and showed that both of these levels were increased.

**Figure 8:**
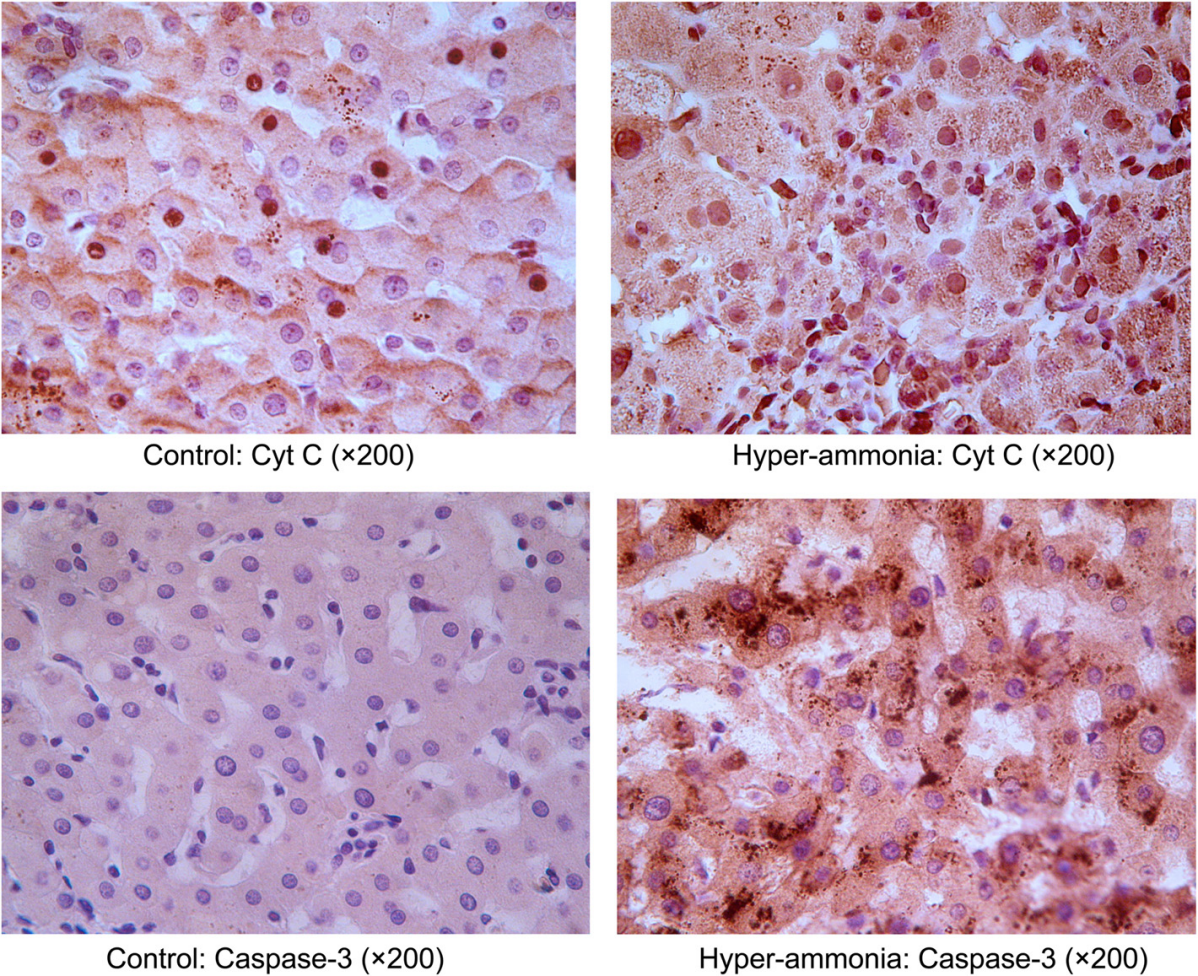
Expression of caspase-3 and Cyt C in hepatocytes.

At the present time, Cyt C is generally accepted as a key step in the apoptotic cascade [40,41]. The apoptosome recruits are considered to be the major enzymes in the commitment to apoptosis [42,43]. In our study, we found that the apoptosis rates in the hyperammonia group of primary hepatocytes were significantly increased. To further examine the apoptosis of cells, we detected the levels of caspase-3 and Cyt C by Real-time PCR and Western blot. We observed that both the Cyt C and caspase-3 levels were increased in ammonia treated group compared with the control group (Figures 4 and 5A), indicating ammonia inducing apoptosis and liver damage in hepatocytes. Moreover, we found higher expression levels of caspase-3 and Cyt C in the tissues of acute liver failure patients (Figure 8).

## Conclusions

In summary, based on the model of liver cell damage resulting from NH_4_Cl, the present study has revealed that increased NH_4_Cl may intensify liver injury, in a manner which was specific for concentration and time. The mechanism possibly involves mitochondrial damage through activation of an intrinsic Ca^2+^-independent apoptosis pathway. It appears that therapeutic approaches to inhibit the concentration of NH_4_Cl and rebalance apoptosis might be efficacious in preventing NH_4_Cl-induced liver damage.

## Competing interests

The authors declare that they have no competing interest.

## Authors’ contributions

QCK, contributed to in the conception, study design. JJL, 1) have made substantial contributions to acquisition of data, or analysis and interpretation of data; 2) have been involved in drafting the manuscript or revising it critically for important intellectual content; and 3) have given final approval of the version to be published. ZJY contributed to collection of test samples, interpretation of the data and critical review of the final manuscript version. QYW, DLL, BJ, YBZ, SS, YFW, SSL and LB provided purely technical help. YWY provided writing assistance. All authors read and approved the final version of the manuscript.

## Pre-publication history

The pre-publication history for this paper can be accessed here:

http://www.biomedcentral.com/1471-230X/14/151/prepub

## References

[B1] Ellis A Wendon J Circulatory, respiratory, cerebral, and renal derangements in acute liver failure: pathophysiology and management Semin Liver Dis 1996 16 379 388 10.1055/s-2007-1007251 9027951

[B2] Sandgren EP Palmiter RD Heckel JL Sandgren EP Palmiter RD Heckel JL Daugherty CC Brinster RL Degen JL Complete hepatic regeneration after somatic deletion of an albumin-plasminogen activator transgene Cell 1991 66 245 256 10.1016/0092-8674(91)90615-6 1713128

[B3] Overturf K Al-Dhalimy M Finegold M Grompe M The repopulation potential of hepatocyte populations differing in size and prior mitotic expansion Am J Pathol 1999 155 2135 2143 10.1016/S0002-9440(10)65531-9 10595942 PMC1866923

[B4] Petersen BE Bowen WC Patrene KD Mars WM Sullivan AK Murase N Boggs SS Greenberger JS Goff JP Bone marrow as a potential source of hepatic oval cells Science 1999 284 1168 1170 10.1126/science.284.5417.1168 10325227

[B5] Lagasse E Connors H Al-Dhalimy M Reitsma M Dohse M Osborne L Wang X Finegold M Weissman IL Grompe M Purified hematopoietic stem cells can differentiate into hepatocytes in vivo Nat Med 2000 6 1229 1234 10.1038/81326 11062533

[B6] Bohm F Regulation of liver regeneration by growth factors and cytokines EMBO Mol Med 2010 2 294 305 10.1002/emmm.201000085 20652897 PMC3377328

[B7] Taub R Liver regeneration: from myth to mechanism Nat Rev Mol Cell Biol 2004 5 836 847 10.1038/nrm1489 15459664

[B8] Lange CM Bojunga J Hofmann WP Wunder K Mihm U Zeuzem S Sarrazin C Severe lactic acidosis during treatment of chronic hepatitis B with entecavir in patients with impaired liver function Hepatology 2009 50 2001 2006 10.1002/hep.23346 19937695

[B9] Widmer R Kaiser B Engels M Wunder K Mihm U Zeuzem S Sarrazin C Hyperammonemia causes protein oxidation and enhanced proteasomal activity in response to mitochondria-mediated oxidative stress in rat primary astrocytes Arch Biochem Biophys 2007 464 1 11 10.1016/j.abb.2007.03.027 17475207

[B10] Felipo V Butterworth RF Neurobiology of ammonia Prog Neurobiol 2002 67 259 279 10.1016/S0301-0082(02)00019-9 12207972

[B11] Drolz A Jäger B Wewalka M Saxa R Horvatits T Roedl K Perkmann T Zauner C Kramer L Ferenci P Fuhrmann V Clinical impact of arterial ammonia levels in ICU patients with different liver diseases Intensive Care Med 2013 39 1227 1237 10.1007/s00134-013-2926-8 23636826

[B12] Skowronska M Albrecht J Alterations of blood brain barrier function in hyperammonemia: an overview Neurotox Res 2012 21 236 244 10.1007/s12640-011-9269-4 21874372 PMC3246587

[B13] Rao KV Norenberg MD Cerebral energy metabolism in hepatic encephalopathy and hyperammonemia Metab Brain Dis 2001 16 67 78 10.1023/A:1011666612822 11726090

[B14] Rao VL Murthy CR Butterworth RF Glutamatergic synaptic dysfunction in hyperammonemic syndromes Metab Brain Dis 1992 7 1 20 10.1007/BF01000437 1608363

[B15] Hazell AS Butterworth RF Hepatic encephalopathy: An update of pathophysiologic mechanisms Proc Soc Exp Biol Med 1999 222 99 112 10.1046/j.1525-1373.1999.d01-120.x 10564534

[B16] Kim IK Niemi AK Krueger C Bonham CA Concepcion W Cowan TM Enns GM Esquivel CO Liver transplantation for urea cycle disorders in pediatric patients: a single-center experience Pediatr Transplant 2013 17 158 167 10.1111/petr.12041 23347504

[B17] Adeva MM Ammonium metabolism in humans Metabolism 2012 61 1495 1511 10.1016/j.metabol.2012.07.007 22921946

[B18] Oka M Maeda S Koga N Kato K Saito T A modified colorimetric MTT assay adapted for primary cultured hepatocytes: application to proliferation and cytotoxicity assays Biosci Biotechnol Biochem 1992 56 1472 1473 10.1271/bbb.56.1472 1368954

[B19] Li Volti G Wang J Traganos F Kappas A Abraham NG Differential effect of heme oxygenase-1 in endothelial and smooth muscle cell cycle progression Biochem Biophys Res Commun 2002 296 1077 1082 10.1016/S0006-291X(02)02054-5 12207883

[B20] Chastre A Jiang W Desjardins P Butterworth RF Ammonia and proinflammatory cytokines modify expression of genes coding for astrocytic proteins implicated in brain edema in acute liver failure Metab Brain Dis 2010 25 17 21 10.1007/s11011-010-9185-y 20217200

[B21] Jayakumar AR Panickar KS Murthy CR Norenberg MD Oxidative stress and mitogen-activated protein kinase phosphorylation mediate ammonia-induced cell swelling and glutamate uptake inhibition in cultured astrocytes J Neurosci 2006 26 4774 4784 10.1523/JNEUROSCI.0120-06.2006 16672650 PMC6674149

[B22] Adams LA Angulo P Lindor KD Nonalcoholic fatty liver disease CMAJ 2005 172 899 905 10.1503/cmaj.045232 15795412 PMC554876

[B23] Hassell T Glevae S Butler M Growth inhibition in animal cell culture Appl Biochem Biotechnol 1991 30 29 41 10.1007/BF02922022 1952924

[B24] Brusilow SW Koehler RC Traystman RJ Cooper AJ Astrocyte glutamine synthetase: importance in hyperammonemic syndromes and potential target for therapy Neurotherapeutics 2010 7 452 470 10.1016/j.nurt.2010.05.015 20880508 PMC2975543

[B25] Garcovich M Zocco MA Roccarina D Ponziani FR Gasbarrini A Prevention and treatment of hepatic encephalopathy: focusing on gut microbiota World J Gastroenterol 2012 18 6693 6700 10.3748/wjg.v18.i46.6693 23239905 PMC3520156

[B26] Jones EA Mullen KD Theories of the pathogenesis of hepatic encephalopathy Clin Liver Dis 2012 16 7 26 10.1016/j.cld.2011.12.010 22321462

[B27] Atluri DK Prakash R Mullen KD Pathogenesis, diagnosis, and treatment of hepatic encephalopathy J Clin Exp Hepatol 2011 1 77 86 10.1016/S0973-6883(11)60126-6 PMC3940085 25755319

[B28] Kala G Kumarathasan R Peng L Leenen FH Hertz L Stimulation of Na+, K + -ATPase activity, increase in potassium uptake, and enhanced production of ouabain-like compounds in ammonia-treated mouse astrocytes Neurochem Int 2000 36 203 211 10.1016/S0197-0186(99)00117-5 10676854

[B29] Xue Z Li B Gu L Hu X Li M Butterworth RF Peng L Increased Na, K-ATPase alpha2 isoform gene expression by ammonia in astrocytes and in brain in vivo Neurochem Int 2010 57 395 403 10.1016/j.neuint.2010.04.014 20447429

[B30] Jia B Zujiang Y Zhenfeng D Lü XQ Li JJ Liu XR Sun R Gao XJ Wang YF Yan JY Kan QC Hyperammonaemia induces hepatic injury with alteration of gene expression profiles Liver Int 2014 34 5 748 758 10.1111/liv.12365 24134218

[B31] BERNARDI P FORTE M The mitochondrial permeability transition pore Novartis Found Symp 2007 287 157 164 discussion 64-9 18074637

[B32] Bernardi P The mitochondrial permeability transition pore: a mystery solved? Front Physiol 2013 4 95 23675351 10.3389/fphys.2013.00095 PMC3650560

[B33] Julie NL Julie IM Kende AI Wilson GL Mitochondrial dysfunction and delayed hepatotoxicity: another lesson from troglitazone Diabetologia 2008 51 2108 2116 10.1007/s00125-008-1133-6 18726085

[B34] Kessova IG Cederbaum AI Mitochondrial alterations in livers of Sod1-/- mice fed alcohol Free Radic Biol Med 2007 42 1470 1480 10.1016/j.freeradbiomed.2007.01.044 17448893 PMC1924491

[B35] Kim JH Choi S Jung JE Roh EJ Kim HJ Capacitative Ca2+ entry is involved in regulating soluble amyloid precursor protein (sAPPalpha) release mediated by muscarinic acetylcholine receptor activation in neuroblastoma SH-SY5Y cells J Neurochem 2006 97 245 254 10.1111/j.1471-4159.2006.03734.x 16524374

[B36] Jang YJ Ryu HJ Choi YO Kim C Leem CH Park CS Improvement of insulin sensitivity by chelation of intracellular Ca(2+) in high-fat-fed rats Metabolism 2002 51 912 918 10.1053/meta.2002.33351 12077741

[B37] Wu G Morris SM Jr Arginine metabolism: nitric oxide and beyond Biochem J 1998 336 1 17 9806879 10.1042/bj3360001 PMC1219836

[B38] Lowenstein CJ Dinerman JL Snyder SH Nitric oxide: a physiologic messenger Ann Intern Med 1994 120 227 237 10.7326/0003-4819-120-3-199402010-00009 8273987

[B39] Yamaguchi M The anti-apoptotic effect of regucalcin is mediated through multisignaling pathways Apoptosis 2013 18 1145 1153 10.1007/s10495-013-0859-x 23670020 PMC3775152

[B40] Huttemann M Pecina P Rainbolt M Sanderson TH Kagan VE Samavati L Doan JW Lee I The multiple functions of cytochrome c and their regulation in life and death decisions of the mammalian cell: From respiration to apoptosis Mitochondrion 2011 11 369 381 10.1016/j.mito.2011.01.010 21296189 PMC3075374

[B41] Wang X The expanding role of mitochondria in apoptosis Genes Dev 2001 15 2922 2933 11711427

[B42] Green DR Apoptotic pathways: paper wraps stone blunts scissors Cell 2000 102 1 4 10.1016/S0092-8674(00)00003-9 10929706

[B43] Li K Li Y Shelton JM Richardson JA Spencer E Chen ZJ Wang X Williams RS Cytochrome c deficiency causes embryonic lethality and attenuates stress-induced apoptosis Cell 2000 101 389 399 10.1016/S0092-8674(00)80849-1 10830166

